# Bonding Performance at the Interface of Glass Fiber-Reinforced Polymer Anchors and Polymer Concrete

**DOI:** 10.3390/polym17192714

**Published:** 2025-10-09

**Authors:** Kai Liu, Wenchao Li, Tianlong Ling, Bo Huang, Meihong Zhou

**Affiliations:** 1 State Key Laboratory of Intelligent Construction and Healthy Operation and Maintenance of Deep Underground Engineering, China University of Mining and Technology, Xuzhou 221116, China; 2 College of Civil and Architectural Engineering, Taishan University, Tai’an 271021, China; liwch@tsu.edu.cn (W.L.); lingtianlong@tsu.edu.cn (T.L.); hbo1987@tsu.edu.cn (B.H.); mhzhou042@tsu.edu.cn (M.Z.); 3 Shandong Engineering Research Center of High-Durability and Corrosion-Resistant New Building Materials, Tai’an 271021, China

**Keywords:** GFRP anchor rod, polymer concrete, pull-out test, bonding performance

## Abstract

Currently, resin polymer anchoring agents are widely used for bolting support in coal mine roadways to anchor the bolts to the surrounding rock mass. However, due to the relatively low strength of the resin anchoring agent itself, the required anchoring length tends to be excessively long. Based on this, this paper proposes the use of resin concrete as a replacement for resin. Compared to resin anchoring agents, resin concrete offers greater mechanical interlocking force with anchor rods, which can reduce the theoretical anchoring length. To systematically investigate the influence of factors such as the diameter and anchorage length of Glass Fiber-Reinforced Polymer (GFRP) bolt on the bond behavior between GFRP bolts and resin concrete, 33 standard pull-out tests were designed and conducted in accordance with the CSA S807-19 standard. Taking the 18 mm-diameter bolt as an example, when the bond lengths were 2D, 3D, 4D, and 5D, the average bond strengths were 41.32 MPa, 39.18 MPa, 38.84 MPa, and 37.44 MPa, respectively. This represents a decrease of 5.18%, 6.00%, and 9.39% for each subsequent increase in bond length. The results indicate that the bond strength between GFRP anchors and resin decreases as the anchorage length increases. Due to the shear lag effect, the average bond strength also decreases with increasing anchor diameter. Taking a 5D (where D is the anchor diameter) anchorage length as a reference, the average bond strengths for anchor diameters of 18 mm, 20 mm, 22 mm, and 24 mm were 37.44 MPa, 33.97 MPa, 32.18 MPa, and 31.50 MPa, respectively. The corresponding reductions compared to the 18 mm diameter case were 9.27%, 14.05%, and 15.87%. Based on the experimental results, this paper proposes a bond–slip constitutive model between the bolt and resin concrete, which consists of a rising branch, a descending branch, and a residual branch. A differential equation relating shear stress to displacement was established, and the functions describing the variation in displacement, normal stress, and shear stress along the position were solved for the ascending branch. Although an analytical solution for the differential equation of the descending branch was not obtained, it will not affect the subsequent derivation of the theoretical anchorage length for the GFRP bolt–resin concrete system, as structural components in practical engineering are not permitted to undergo excessive bond-slip.

## 1. Introduction

Bolt structures have gained widespread application in various engineering projects, including mines, foundation pits, slopes, underground works, reservoir dams, marine engineering, and airports, as well as anti-overturning and seismic-resistant structures, owing to their high reliability and cost-effectiveness. However, practical experience with bolt structures has revealed a significant concern: widespread corrosion of steel bolts is prevalent, primarily because many of these structures are situated in complex geological or harsh climatic environments. This raises serious doubts regarding their long-term safety and durability. Although anti-corrosion measures—such as hot-dip galvanizing, epoxy resin coatings, centralizers for bolts, and adding anti-corrosion agents to grout—have been employed in anchoring engineering, these methods have consistently failed to provide a fundamental solution to the problem [1]. Under these circumstances, the use of non-metallic material bolts has emerged as the optimal alternative.

Existing research findings indicate that using Fiber-Reinforced Polymer (FRP) bars as a replacement for steel bars in anchor systems is one of the effective solutions to address the issue of anchor corrosion [2,3]. Research on their application in geotechnical anchoring has become a hot topic in FRP studies since the 21st century. After conducting field comparative tests on slopes reinforced with Glass Fiber-Reinforced Polymer (GFRP) anchors, Carbon Fiber-Reinforced Polymer (CFRP) anchors, and steel anchors, Cheng et al. [4] concluded that CFRP anchors are not suitable as geotechnical reinforcement materials due to their high cost, low shear strength, and poor interfacial bonding performance with grouting material. The differences in mechanical properties between GFRP anchors and steel bars result in significantly distinct bond behaviors in the GFRP anchor–concrete interface compared to the steel bar–concrete interface. Numerous scholars have conducted extensive experimental studies on the bond behavior between GFRP bars and concrete [5,6,7,8,9,10,11]. Based on these investigations, constitutive models for the bond–slip relationship between GFRP bars and ordinary concrete have been established, and empirical formulas for determining the basic anchorage length have been proposed [12,13].

Resin anchorage agents possess relatively low strength and excessive anchorage length is often required. Using resin concrete as a replacement for resin anchorage agents can provide greater mechanical interlock, thereby reducing the theoretical anchorage length and achieving more reliable anchorage between the resin concrete, the anchor bar, and the surrounding rock. The superior corrosion resistance of FRP bars compared to steel bars enables their potential use in combination with various special types of concrete, which has also become a research focus in recent years. Scholars have conducted studies on the bond performance between FRP bars and a variety of special concretes, including coral concrete, recycled aggregate concrete, steam-cured concrete, and rubber concrete [14,15,16,17,18,19,20]. However, theoretical research in this area remains limited, and studies on the direct bond behavior between resin concrete and GFRP anchors are particularly scarce. Existing research indicates that resin concrete differs significantly from ordinary concrete in terms of aggregate characteristics, mechanical properties, and physico-chemical characteristics [21,22]. Consequently, the bond performance between resin concrete and GFRP anchors requires further in-depth investigation. Furthermore, in engineering applications, insufficient anchorage length of GFRP anchors may lead to reduced support stiffness or even bond failure. Therefore, accurate theoretical analysis and calculation of anchorage length fundamentally depend on comprehensive experimental research and theoretical analysis of the bond mechanism between the two materials.

Based on existing bond–slip constitutive models for GFRP bars in concrete [23,24,25], this study develops a continuous curve model applicable to resin concrete through theoretical derivation, compares it with experimental data, and subsequently derives an analytical solution for the bond–slip differential equation. This solution theoretically characterizes the distributions of slip, bond stress, and bolt stress along the anchorage length, thereby providing a theoretical basis for engineering design.

## 2. Experimental Investigation

### 2.1. Test Materials

(1) Raw Materials for Resin Concrete (UPC)

The quartz sand aggregate used in this experiment was supplied by Huarong Quartz Sand Factory, Jinan, Shandong, China, with particle sizes ranging from 120 to 20 mesh (3–5 mm). The resin matrix consisted of Type 9611 unsaturated polyester resin(New Solar Technology Group Co., Ltd., Changzhou, Jiangsu, China), a linear polymer formed through polycondensation of saturated dibasic acids, unsaturated dibasic acids, and dihydric alcohols, subsequently diluted with crosslinking monomers or reactive solvents to form a viscous resin solution. The tensile strength is 56 MPa, and the elastic modulus is 3.2 GPa.

The formulation included Methyl Ethyl Ketone Peroxide (MEKP) V388 curing agent and 3-(Trimethoxysilyl) propyl methacrylate (MPS) KH570 (New Solar Technology Group Co., Ltd., Changzhou, Jiangsu, China)coupling agent, added at 1% and 1.5% of the resin weight, respectively.

(2) Preparation and Mechanical Properties of UPC Specimens

Following the mix proportions in Table 1, the curing agent and coupling agent were first mixed into the resin. Quartz sand was added and stirred until homogeneous after the resin mixture turned milky white. Specimens were prepared in accordance with GB/T 50081-2019 [26] (Standard for Test Methods of Mechanical Properties of Concrete). After 7 days of natural curing, the standard cube compressive strength was measured at 98.22 MPa, while the splitting test yielded a characteristic tensile strength value of 4.97 MPa.

(3) Mechanical Properties of GFRP Bolts

**Figure 1:**
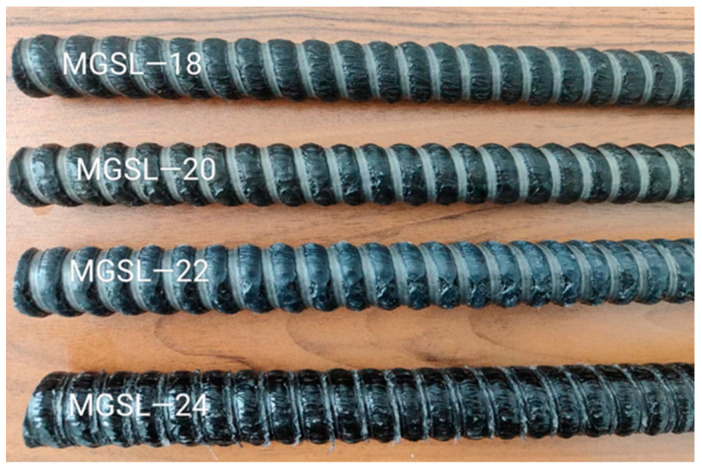
GFRP bolts with different diameters used in the experiment.

The GFRP bolts (Shandong Safety Industries Co.,Ltd.,Tai’an, Shandong, China) used in this study primarily composed of vinyl ester resin and alkali-resistant glass fibers. The benchmark group consisted of 18 mm-diameter ribbed-surface GFRP bolts. The performance test was conducted using a WAW-1000D electro-hydraulic servo universal testing machine(Jinan Fangyuan Experimental Instrument Co., Ltd., Jinan, Shandong, China) which exhibited the following mechanical characteristics (average values of a total of 10 GFRP anchor specimens): tensile strength: 710 MPa, elastic modulus: 53.85 GPa, and elongation at break: 2.02%. Control groups included GFRP bolts with diameters of 20 mm, 22 mm, and 24 mm. The surface morphology of all test bolts is shown in Figure 1, where MGSL means Mining anchor Glass Fiber Screw-threaded Resin.

### 2.2. Specimen Preparation

The experiment utilized 150 mm × 150 mm × 150 mm demountable plastic molds(Jinan Kehui Experimental Equipment Co., Ltd., Jinan, Shandong, China). Considering factors including the diameter of the PVC sleeve, anchor rod diameter, and concrete cover thickness, holes were drilled on both sides of the mold. The specimen preparation procedure was as follows:

(1) Mold Preparation and Rod Installation

A mold release agent was applied to the inner surfaces of the mold. GFRP rods were cut to the specified lengths in accordance with standard GB/T 50081-2019 [26] and inserted through the pre-formed holes in the mold. PVC sleeves of appropriate length were selected based on the required bond length and slid onto the loading end of each GFRP rod. The interface between the GFRP rods and the PVC sleeves was sealed with hot-melt adhesive to prevent concrete infiltration.

(2) Concrete Placement and Curing

Mold holes were sealed with hot-melt adhesive to prevent resin leakage. Mixed resin concrete(Tai’an Zhonglian Concrete Co., Ltd., Tai’an, Shandong, China)was poured into molds and compacted on a vibration table. The mold was removed after 24 h of natural curing in air (20 ± 3 °C). We monitored mold surface temperature by hand-delayed demolding if significant heat generation was detected.

(3) Anchorage System Installation

**Figure 2:**
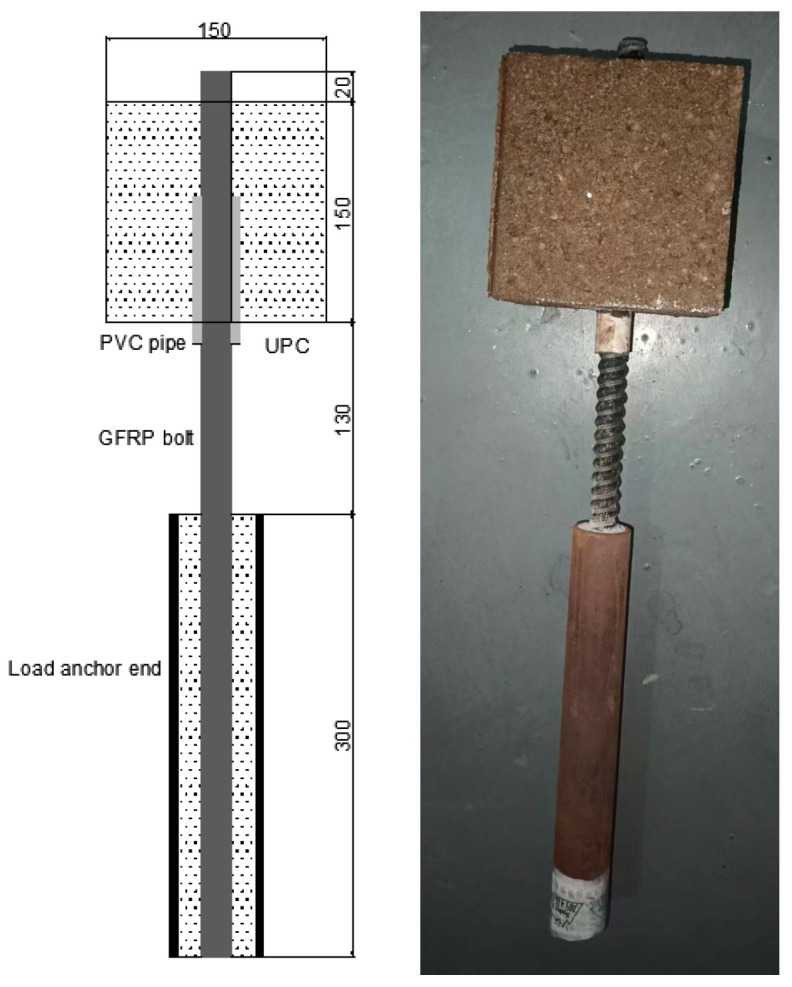
Specimens for GFRP bolt-UPC bond tests.

To prevent the possible damage of GFRP rod during testing, high-strength grouted steel tubes (Jinan Kehui Experimental Equipment Co., Ltd., Jinan, Shandong, China)were installed at the loading ends. After curing for 7 days, test specimen were ready for testing. (Figure 2 shows finished test specimens).

### 2.3. Experimental Testing

The bond performance test was conducted using a WAW-1000D electro-hydraulic servo universal testing machine with the following steps in accordance with CSA S807-19 [27]:

(1) Fixture Installation

The upper clamping head of the custom test fixture was securely fastened to the testing machine’s actuator. The fixture was aligned to ensure pure axial loading without eccentricity.

(2) Specimen Mounting

The prepared GFRP bolt–resin concrete specimen was carefully inserted through the fixture base plate. The specimen was then bolted to the fixture using high-strength fasteners to prevent slippage.

(3) Loading Configuration

The lower hydraulic grip of the testing machine clamped the steel transfer tube connected to the GFRP bolt.

(4) Displacement Measurement

Two precision dial gauges (0.001 mm resolution) (Jinan Kehui Experimental Equipment Co., Ltd., Jinan, Shandong, China)were installed to measure the following:

Free-length bolt displacement (positioned 50 mm from the concrete surface).

Resin concrete block movement (mounted on a stable reference frame).

Data acquisition was synchronized with the loading system.

(5) Pre-Test Verification

**Figure 3:**
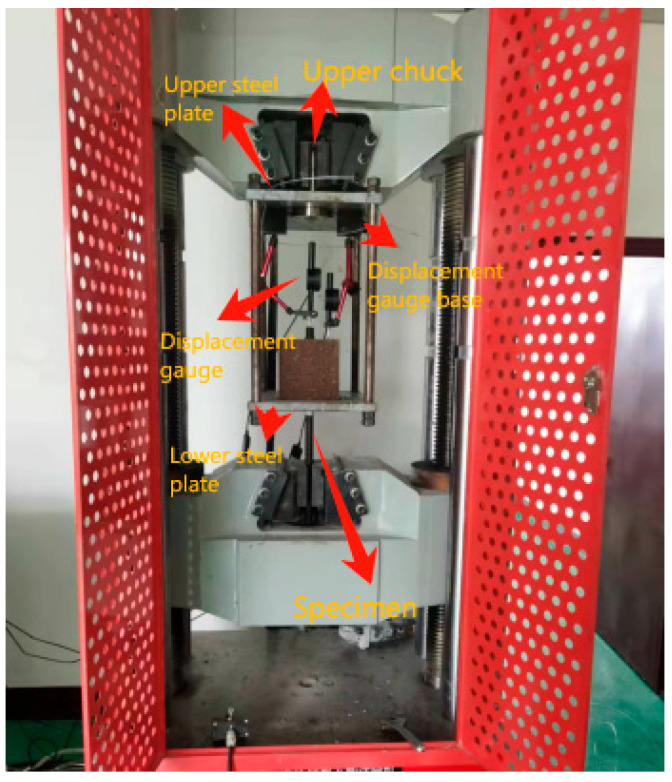
Schematic diagram of the experimental setup.

A 5 kN pre-load was applied to confirm proper seating. All instruments were zeroed and calibrated. The final test setup is illustrated in Figure 3.

The bond performance test was conducted under displacement control at 0.2 mm/min in accordance with CSA S807-19 [27]. The test was terminated upon meeting any of the following conditions:

(1) Bolt Fracture

The GFRP anchor rod ruptures due to tensile overload.

(2) Concrete Splitting Failure

The resin concrete block cracks or splits under bond stress.

(3) Bond Failure (Pull-out)

The GFRP bolt completely detaches from the resin concrete.

(4) Excessive Slip (20 mm)

The relative slip between the free-end bolt segment and the concrete exceeds 20 mm, indicating bond degradation.

## 3. Bond–Slip Relationship Curve Model

### 3.1. Experimental Results

Thirty-three GFRP anchor-UPC pull-out specimens were tested to investigate the influence of bar diameter and bond length on bond strength. The failure modes included pull-out failure, GFRP anchor fracture failure, and UPC splitting.

Upon splitting the pull-out failure specimens, it was observed that the ribs on the surface of the GFRP anchors in the bonded section were severely worn, with some areas ground flat. The internal fibers of the GFRP anchors were exposed, and a large amount of residual material from the GFRP anchors adhered to the concrete.

For the GFRP anchor fracture failure specimens, cracks appeared in the non-bonded section of the anchor, leading to a rapid drop in test load. This type of failure mostly occurred in specimens where the GFRP anchor exhibited higher bond strength. Upon splitting the failed specimens, it was observed that the sand coating on the bonded section of the GFRP anchor had significantly peeled off, while the ribs were not completely worn flat.

UPC splitting failure manifested as a sudden brittle fracture of the test block, with aggregate breakage and only a small amount of anchor surface fibers adhering to the concrete.

**Figure 4:**
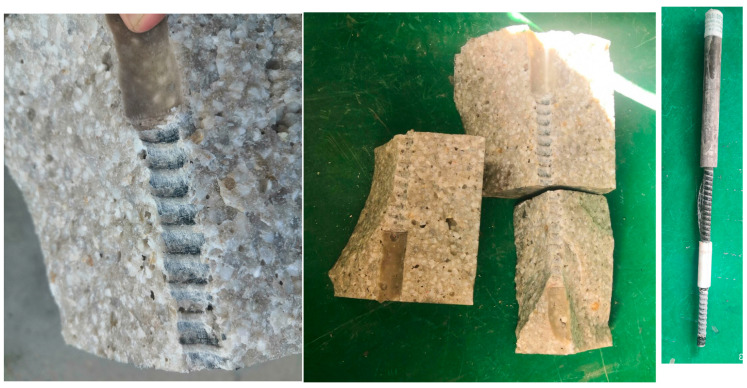
Field photos of specimen failure.

The three failure modes are illustrated in Figure 4.

Two dial gauges were used to measure the absolute displacement of the free-end anchor (Sf) and the absolute displacement of the free-end concrete (Sc), respectively. The difference between the two values represents the relative sliding displacement between the GFRP anchor and the resin concrete specimen at the free end. The data from the dial gauges were collected using a micrometer signal acquisition device. In this study, it was initially assumed that shear stress was uniformly distributed within the bonded segment. The average bond strength of the specimens was calculated using a formula, and thus, the bond strength was determined according to Equation (1).

(1) $$\tau =\frac{P}{\pi DL}$$

where

*τ* is the average bond strength of the specimen (MPa);

*P* is the ultimate pull-out load of the specimen (N);

*d* is the diameter of the GFRP anchor bar (mm);

*L* is the bonded length of the GFRP anchor bar in the concrete (mm).

The experimental data are presented in Table 2. The labels in the table sequentially indicate the anchor type, diameter, and bonded length.

For example, GFRP-18-3D represents a GFRP anchor bar with a diameter of 18 mm and a bonded length of 54 mm (where 3D denotes three times the diameter).

The following analyzes the main factors affecting bond strength.

(1) Influence of Bond Length on Bond Strength

**Figure 5:**
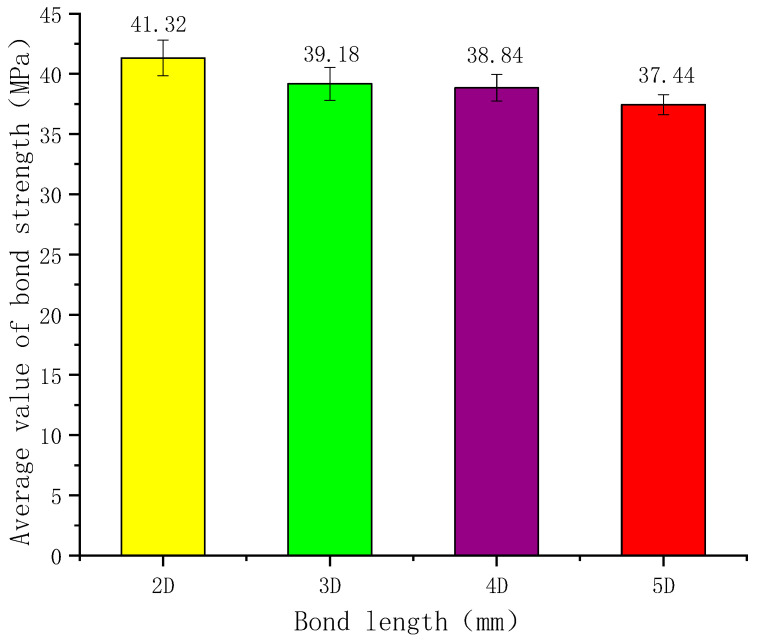
Bond strength of 18 mm diameter anchor rod varies with bond length.

Taking an 18 mm diameter anchor rod as an example, when the bond lengths are 2D, 3D, 4D, and 5D, the average bond strengths are 41.32 MPa, 39.18 MPa, 38.84 MPa, and 37.44 MPa, respectively. The latter values decreased by 5.18%, 6.00%, and 9.39% compared to the former. The variation in average bond strength with bond length is shown in Figure 5. The bond strength between GFRP anchor rods and UPC decreases as the bond length increases. Because of the shear lag phenomenon, a longer bond length leads to a more uneven distribution of bond stress, resulting in a smaller ratio of the average bond strength at failure to the actual maximum bond strength [28]. This is the primary reason why bond strength decreases with increasing bond length. The reduction rate indicates that bond length is a significant factor influencing bond strength. However, beyond a certain point, the decrease in average bond strength becomes relatively small [28].

Test results indicate that the failure modes of GFRP anchor rods and resin concrete primarily manifest in three forms: tensile fracture of the anchor rod, splitting failure of the concrete, and pull-out failure due to anchorage loss.

(2) Influence of Anchorage Diameter on Bond Strength

The surface of GFRP anchor rods is uneven, and their bond strength with resin concrete mainly stems from mechanical interlocking. During initial loading, when shear stress begins to act on the interface between the GFRP anchor rod and the resin concrete, chemical adhesion and frictional resistance play the primary roles. As interfacial shear stress increases, the contribution of chemical adhesion and friction gradually diminishes or disappears, while mechanical interlocking becomes dominant.

During the loading process, bond–slip first occurs at the loading end, and internal cracks develop in the concrete, propagating outward from the surface of the GFRP anchor rod. Due to the low tensile strength of deep-ribbed GFRP anchor rods and their strong interaction with the concrete anchorage, they fracture before rapid crack expansion, resulting in tensile failure. When cracks extend to the concrete surface and penetrate it, the concrete splits completely into 2–3 parts, leading to full splitting failure.

For GFRP anchor rods with a smaller diameter and a relatively larger concrete cover thickness (ratio of cover thickness to anchor rod diameter), if the anchorage length is insufficient, the anchor rod may gradually pull out before cracks penetrate the concrete cover. Meanwhile, cracks may propagate through the cover but not fully split the specimen, resulting in a splitting-pull-out failure. In such cases, residual mortar remains between the ribs of the GFRP anchor rod, and minor spalling is observed on the rib surfaces, but no severe damage occurs.

**Figure 6:**
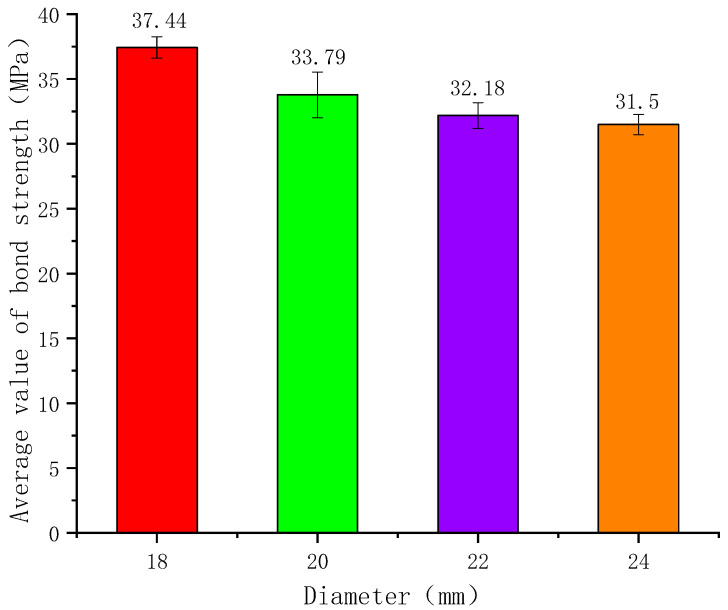
Relationship between average bond strength and anchor rod diameter.

Taking an anchorage length of 5D (where D is the anchor rod diameter) as a reference, the average bond strengths for anchor rod diameters of 18 mm, 20 mm, 22 mm, and 24 mm were 37.44 MPa, 33.97 MPa, 32.18 MPa, and 31.50 MPa, respectively. Compared to the 18 mm diameter, the bond strengths decreased by 9.27%, 14.05%, and 15.87% for the larger diameters, as illustrated in Figure 6.

As clearly shown in the figure, the ultimate average bond stress of GFRP anchors decreases with increasing diameter, which can be attributed to the Poisson effect and shear lag effect. During loading, the Poisson effect causes the GFRP anchor to undergo axial elongation while simultaneously reducing in diameter. The larger the anchor diameter, the more pronounced the radial contraction becomes. This results in lower contact pressure at the interface between the anchor and concrete, thereby reducing the frictional resistance and mechanical interlock at the bond interface between the GFRP anchor and resin concrete, ultimately leading to a decrease in bond strength.

Additionally, shear lag occurs in the GFRP anchor rod, leading to significantly greater deformation at the outer edges than at the center of the cross-section. This results in non-uniform stress distribution across the rod. While the bond strength of the GFRP anchor rod depends on the maximum bond stress at the edges, only the average bond stress can be measured experimentally. As the diameter increases, the shear lag effect becomes more pronounced, further reducing the calculated average bond strength.

**Figure 7:**
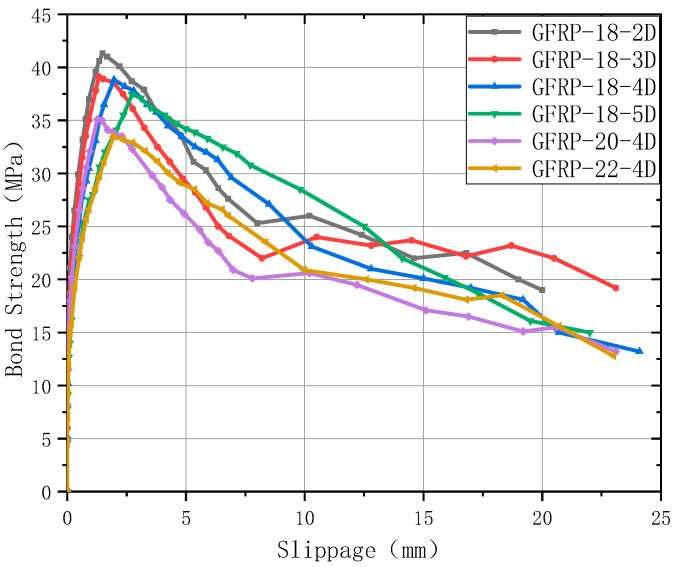
Bond–slip curve of pull-out failure specimen.

The bond-slip curve of pull-out failure specimen is presented in Figure 7, which provided critical reference data for subsequent model analysis.

### 3.2. Bond–Slip Constitutive Model Proposed in This Study

Given the unique properties of resin concrete, traditional models are not entirely suitable for characterizing the bond behavior between GFRP anchor rods and resin concrete. Based on experimental investigations of the bond–slip behavior of GFRP anchor rods in resin concrete, the following bond–slip constitutive model is proposed:

(2) $$\mathrm{Rising branch}\;\tau /\tau_{1}=(s/s_{1}{)}^{0.3}\;s\leq s_{1}$$

(3) $$\mathrm{Declining branch}\;\tau /\tau_{1}=1-0.09(s/s_{1}-1)\;s_{1}\leq s\leq s_{3}$$

(4) $$\mathrm{Residual branch}\;\tau =\tau_{3}\;s\geq s_{3}$$

In the Equation, $\tau_{1}$ and $s_{1}$ are the peak bond stress and the corresponding slip.

In practical engineering applications (e.g., when calculating the anchorage length of bolts), only the ascending branch is considered, while the descending branch and residual branch are typically neglected [29,30]. Thus, this model is divided into just three segments. The model features clear physical concepts and a simple, intuitive formulation, while also satisfying the conditions of an infinite initial slope and continuous smoothness at extremum points.

### 3.3. Comparison Between Theoretical and Experimental Values

**Figure 8:**
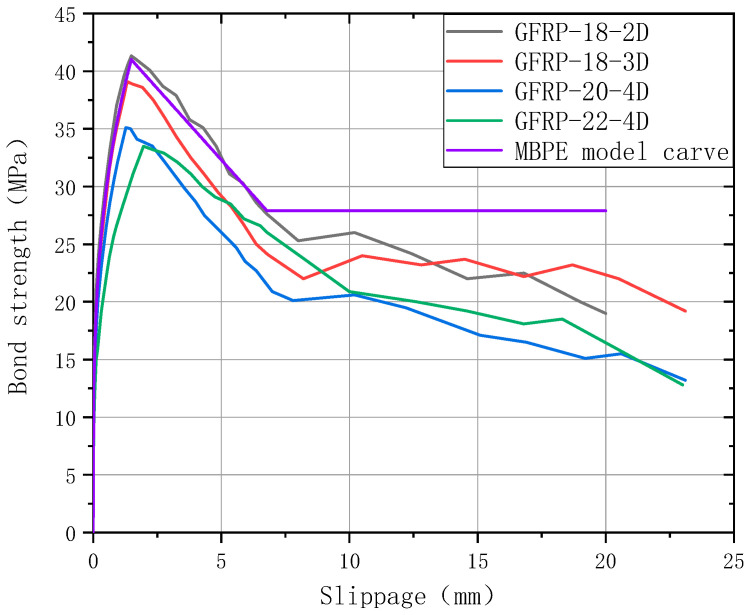
Comparison of theoretical curve and experimental curve.

Figure 8 presents a comparative analysis between the experimental bond strength-displacement curves of different groups and the theoretical predictions from the MBPE model.

**Figure 9:**
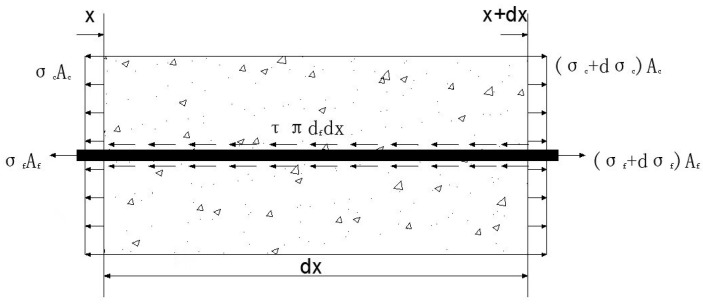
Stress of a basic differential unit.

Taking a differential unit element for analysis, its stress state is shown in Figure 9.

① Equilibrium Equation:

Taking the entire system as the research object, in this case the bonding force between the GFRP anchor rod and concrete acts as an internal force.

(5) $$A_{c}d\sigma_{c}+{\;A}_{f}d\sigma_{f}=\;0$$

The study focuses on GFRP anchor bars. The stress difference at both ends of the anchor bar is balanced by the longitudinal shear stress on its surface, which constitutes the bond stress provided by the surrounding concrete.

(6) $$A_{f}d\sigma_{f\;}=\pi d_{f}\tau \left(x\right)\;\mathrm{dx}\;$$

(7) $$i.e.,\;\frac{\pi d_{f}^{2}}{4}d\sigma_{f\;}=\pi d_{f}\tau \left(x\right)\;\mathrm{dx}\;$$

② Constitutive equation in the elastic stage:

(8) $$\sigma_{c}=E_{c}\varepsilon_{c}\;,\sigma_{f}=E_{f}\varepsilon_{f}$$

③ Deformation compatibility equation:

(9) $$s(x)=s_{\mathrm{ff}}(x)+\int \left(\varepsilon_{f}-\varepsilon_{c}\right)\;\mathrm{dx}$$

From a physical perspective, the relative slip s(x) at any given point represents the difference in displacement between the GFRP anchor bar and the surrounding resin concrete, expressed as:

(10) $${s(x)\;=\;s}_{f}\left(x\right)-s_{c}\left(x\right)$$

where

A_f_ and A_c_, respectively are the cross-sectional areas of the GFRP anchor rod and the resin concrete;

σ_c_ and ε_c_, respectively, are the normal stress and strain of concrete;

σ_f_ and ε_f_, respectively, are the normal stress and strain of GFRP anchor bars;

τ(x) is the average bond stress at this element;

D_f_ is the diameter of GFRP anchor bars;

E_f_ is the elastic modulus of GFRP anchor bars;

E_c_ is the elastic modulus of resin concrete;

s_ff_(x) is free-end slip displacement of the pull-out specimen.

Differential equation (8):

(11) $$\frac{\mathrm{ds}}{\mathrm{dx}}=\frac{{\mathrm{ds}}_{f}}{\mathrm{dx}}-\frac{{\mathrm{ds}}_{c}}{\mathrm{dx}}=\varepsilon_{f}\left(x\right)-\varepsilon_{c}\left(x\right)$$

Differentiate the above equation again and substitute the constitutive Equation (7),

(12) $$\frac{d^{2}s}{{\mathrm{dx}}^{2}}=\frac{d\sigma_{f}}{E_{f}\mathrm{dx}}-\frac{d\sigma_{c}}{E_{c}\mathrm{dx}}$$

From Equation (5), the stress relationship between the GFRP anchor rod and resin concrete can be expressed as

(13) $$d\sigma_{c}=-\frac{A_{f}}{A_{c}}d\sigma_{f}\;$$

From Equation (7), the relationship between the stress in the GFRP anchor rod and the interfacial bond stress can be expressed as,

(14) $$\frac{d\sigma_{f\;}}{\mathrm{dx}}=\frac{4}{d_{f}}\tau \left(x\right)$$

Substituting Equations (13) and (14) into Equation (12), the differential relationship between bond stress and slip can be obtained as

(15) $$\frac{d^{2}s}{{\mathrm{dx}}^{2}}-\frac{4}{d_{f}}\left(\frac{1}{E_{f}}+\frac{A_{f}}{A_{c}E_{c}}\right)\tau \left(x\right)=0$$

Let $\frac{4}{d_{f}}\left(\frac{1}{E_{f}}+\frac{A_{f}}{A_{c}E_{c}}\right)=m$, Equation (15) equal to

(16) $$\frac{d^{2}s}{{\mathrm{dx}}^{2}}-m\tau \left(x\right)=0$$

The above equation is the bond–slip differential equation, which establishes the relationship between the relative slip displacement and bond stress of the GFRP anchor rod and resin concrete. Theoretically, it defines the connection between the relative slip, bond stress, and the variable “x”. However, it should be noted that this expression is derived under purely theoretical conditions. In reality, the mechanical behavior of structural components is not always perfectly ideal. Factors such as material variability, the distribution of GFRP anchor rods within the component, and other practical considerations can influence the bond behavior. Therefore, the actual pull-out failure of GFRP-reinforced concrete components may not fully satisfy the bond–slip differential equation (Equation (1)).

Substitute the proposed model (Equation (2)) into Equation (16)

① Rising branch:

(17) $$\frac{d^{2}s}{{\mathrm{dx}}^{2}}-k_{1}s^{0.3}=0$$

In the equation, $k_{1}=m\tau_{1}/s_{1}^{0.3}$.

To solve for this, let $\frac{\mathrm{ds}}{\mathrm{dx}}=p$, then $\frac{d^{2}s}{{\mathrm{dx}}^{2}}=\frac{dp}{\mathrm{dx}}=\frac{dp}{\mathrm{ds}}\cdot \frac{\mathrm{ds}}{\mathrm{dx}}=p\cdot \frac{dp}{\mathrm{ds}}$

(18) $$p\cdot \frac{dp}{\mathrm{ds}}=k_{1}s^{0.3}$$

After rearranging terms and separating variables, integrate both sides to yield the following:

(19) $$p\cdot dp=k_{1}s^{0.3}\cdot ds$$

(20) $$\int p\cdot \mathrm{dp}=\int k_{1}s^{0.3}\mathrm{ds}$$

(21) $$\mathrm{then:}\;p^{2}=\frac{{2k}_{1}}{1.3}s^{1.3}+c_{1}$$

where c_1_ is the constant of integration.

Apply the boundary conditions:

At x = 0, slip displacement s = 0, $p=\frac{\mathrm{ds}}{\mathrm{dx}}=0$. Substitution formula Equation (18), yields c_1_ = 0, rewrite it as the following:

(22) $$p=\frac{\mathrm{ds}}{\mathrm{dx}}=\sqrt{\frac{k_{1}}{0.65}}s^{0.65}$$

(23) $$\mathrm{dx}=\sqrt{\frac{0.65}{k_{1}}}s^{-0.65}\mathrm{ds}$$

Integrate both sides of the above equation to yield the following:

(24) $$x=\frac{1}{0.35}\sqrt{\frac{0.65}{k_{1}}}s^{0.35}+c_{2}$$

At x = 0, slip displacement s = 0, $p=\frac{\mathrm{ds}}{\mathrm{dx}}=0$, substitute into Equation (24), yields c_2_ = 0. Invert Equation (24) to obtain the slip displacement distribution along the interface.

(25) $$s\left(x\right)={\left(0.35\sqrt{\frac{k_{1}}{0.65}}x\right)}^{\frac{1}{0.35}}$$

Substitute $m=\frac{4}{d_{f}}\left(\frac{1}{E_{f}}+\frac{A_{f}}{A_{c}E_{c}}\right)$ and $k_{1}=m\tau_{1}/s_{1}^{0.3}$ into Equation (25) to yield the following:

(26) $$s\left(x\right)={\left[0.35\sqrt{\frac{6.154}{d_{f}}\left(\frac{1}{E_{f}}+\frac{A_{f}}{A_{c}E_{c}}\right)\frac{\tau_{1}}{s_{1}^{0.3}}}x\right]}^{\frac{1}{0.35}}$$

Equation (26) is the analytical solution of the differential equation, it reflects the variation law of the relative bond–slip displacement “s” with the embedded length “x”. Substituting this equation into Equation (2) yields the variation law of shear stress along the embedded length “x”:

(27) $$\tau \left(x\right)=\frac{0.887\tau_{1}^{1.4285}}{s_{1}^{0.42855}}{\left[\frac{1}{d_{f}}\left(\frac{1}{E_{f}}+\frac{A_{f}}{A_{c}E_{c}}\right)\right]}^{0.4285}x^{0.857}$$

Integrating both sides of Equation (14) and substituting Equation (27) yields the following:

(28) $$\sigma \left(x\right)=\frac{4x}{d_{f}}\cdot \tau \left(x\right)=\frac{3.547\tau_{1}^{1.4285}}{s_{1}^{0.42855}\cdot d_{f}^{1.4285}}{\left(\frac{1}{E_{f}}+\frac{A_{f}}{A_{c}E_{c}}\right)}^{0.4285}x^{1.857}$$

Equations (26)–(28) are the analytical solutions corresponding to the ascending branch of the bond–slip model proposed in this study. Based on these solutions, the slip displacement, bond stress at different embedment depths, and the normal stress on the GFRP anchor rod’s cross-section can be calculated under various load levels.

② The descending branch:

Substituting Equation (3) into Equation (16):

(29) $$\frac{d^{2}s}{{\mathrm{dx}}^{2}}-m\left[1-0.09\left(\frac{s}{s_{1}}-1\right)\right]\tau_{1}=0$$

Let $a=-0.09m\frac{\tau_{1}}{s_{1}}$, $b=1.09m\tau_{1}$,

Revise Equation (29) to

(30) $$\frac{d^{2}s}{dx^{2}}=as+b$$

To solve it, let $\frac{\mathrm{ds}}{\mathrm{dx}}=p$, obtain: $\frac{d^{2}s}{{\mathrm{dx}}^{2}}=\frac{dp}{\mathrm{dx}}=\frac{dp}{\mathrm{ds}}\cdot \frac{\mathrm{ds}}{\mathrm{dx}}=p\cdot \frac{dp}{\mathrm{ds}}$

(31) $$p\cdot \frac{dp}{ds}=as+b$$

After rearranging and separating the variables, integrate both sides:

$$p\cdot dp=\left(as+b\right)\cdot ds$$

$$\int p\cdot dp=\int \left(as+b\right)ds$$

(32) $$\mathrm{yields:}\;\frac{1}{2}p^{2}=\frac{1}{2}as^{2}+bs+c_{1}$$

Among them, c_1_ is the constant of integration.

Applying the boundary conditions:

At x = 0, slip displacement s = 0, $p=\frac{\mathrm{ds}}{\mathrm{dx}}=0$. Substitution Equation (32), yields: c_1_ = 0, Rewrite Equation (32) as

(33) $$p=\frac{ds}{dx}=\sqrt{a}s+2\sqrt{bs}$$

(34) $$dx=\frac{1}{\sqrt{a}s+2\sqrt{bs}}ds$$

Let $\sqrt{bs}=t$, obtain $s=\frac{t^{2}}{b}$

$$ds=\frac{2t}{b}dt$$

$$\mathrm{obtain}\;dx=\int \frac{1}{\sqrt{a}s+2\sqrt{bs}}ds$$

$$dx=\int \frac{1}{\sqrt{a}\frac{t^{2}}{b}+2t}\cdot \frac{2t}{b}dt$$

$$dx=\int \frac{2}{\sqrt{a}t+2b}dt$$

$$dx=\int \frac{2}{\sqrt{a}t+2b}\cdot \frac{1}{\sqrt{a}}d\left(\sqrt{a}t+2b\right)$$

Integrating both sides of the above equation, obtain

(35) $$x=\frac{2}{\sqrt{a}}\ln\left|\sqrt{a}t+2b\right|+c_{2}$$

Substitute $\sqrt{bs}=t$, obtain

(36) $$x=\frac{2}{\sqrt{a}}\ln\left|\sqrt{abs}+2b\right|+c_{2}$$

At x = 0, slip displacement s = 0, $p=\frac{\mathrm{ds}}{\mathrm{dx}}=0$. Substitute Equation (36) to yield the following: $c_{2}=-\frac{2}{\sqrt{a}}\ln\left|2b\right|$.

(37) $$x=\frac{2}{\sqrt{a}}\ln\left|\sqrt{abs}+2b\right|-\frac{2}{\sqrt{a}}\ln\left|2b\right|$$

This equation cannot yield an exact analytical solution. However, in practical engineering applications, structural components are not permitted to undergo excessive bond–slip deformation. Therefore, it will not affect the subsequent derivation of the theoretical anchorage length for GFRP anchor rods in resin concrete.

## 4. Conclusions

In accordance with the CSA S807-19 standard, this study designed and fabricated 33 sets of standard bond test specimens and conducted pull-out tests. It systematically investigated the effects of various factors, including GFRP anchor diameter and anchorage length, on the bond performance between GFRP anchors and resin concrete, leading to the following conclusions:

(1) This study proposes the use of resin concrete to replace resin anchorage agents in coal mines. The results demonstrate that resin concrete provides enhanced mechanical interlock and higher anchorage strength.
(2) The effect of bond length on anchorage strength was investigated; it was found that bond strength decreases with increasing bond length. Taking 18 mm diameter anchors as an example, when the bond lengths were 2D, 3D, 4D, and 5D, the average bond strengths were 41.32 MPa, 39.18 MPa, 38.84 MPa, and 37.44 MPa, respectively. Compared to the preceding shorter bond length, the strengths decreased by 5.18%, 6.00%, and 9.39%, respectively.
(3) The influence of anchor diameter on anchorage strength was also investigated. Due to the shear lag effect, the average bond strength decreases as the GFRP anchor diameter increases. Using a bond length of 5D (where D is the anchor diameter) as a reference, for anchor diameters of 18 mm, 20 mm, 22 mm, and 24 mm, the corresponding average bond strengths were 37.44 MPa, 33.97 MPa, 32.18 MPa, and 31.50 MPa. These values represent decreases of 9.27%, 14.05%, and 15.87% compared to the preceding smaller diameter.
(4) Building on existing studies and tests, a simpler bond–slip model for GFRP anchors in resin concrete is developed. Since engineers mainly care about the rising part of the curve in real applications, our model focuses only on three key stages. It is easy to understand yet meets key requirements like infinite initial slope and smooth peak transition. By establishing and solving the differential equations, analytical expressions for the slip displacement, normal stress, and shear stress distribution along the anchorage length in the ascending branch are derived, providing a theoretical basis for engineering design.

## Figures and Tables

**Table 1:** Mix Proportion of UPC.

Resin	3–5 mm Quartz Sand	20–40 Mesh Quartz Sand	40–70 Mesh Quartz Sand	70–120 Mesh Quartz Sand
1	2.829	1.886	0.943	0.629

**Table 2:** Pull-out test results.

Specimen Number	$F_{\max}$/kN	${\bar{F}}_{\max}$/kN	$\tau_{\max}$/MPa	${\bar{\tau }}_{\max}$/MPa	$S_{r}$/mm	${\bar{S}}_{r}$/mm	Failure Mode
GFRP-18-2D	85.64	84.12	42.07	41.32	1.60	1.49	Pull off
	80.66		39.62		1.52		Pull off
	86.05		42.27		1.36		Pull off
GFRP-18-3D	120.74	119.64	39.54	39.18	1.42	1.33	Pull off
	123.15		40.33		1.67		Pull off
	115.03		37.67		0.89		Pull off
GFRP-18-4D	152.97	158.14	37.57	38.84	1.83	1.97	Pull off
	161.31		39.62		2.11		Pull off
	160.13		39.33		1.98		Pull off
GFRP-18-5D	180.67	180.84	36.51	37.44	4.21	2.73	Rebar fracture
	179.40		37.68		3.43		Rebar fracture
	182.45		38.12		0.55		Rebar fracture, UPC split
GFRP-20-4D	176.48	177.57	35.11	35.33	1.32	1.27	Pull off
	174.87		34.79		1.36		Pull off
	181.36		36.08		1.12		Pull off
GFRP-20-5D	224.69	212.29	35.76	33.79	1.26	1.77	UPC split
	208.66		33.21		1.55		Pull off
	203.51		32.39		2.51		Pull off
GFRP-22-4D	207.77	203.69	34.16	33.49	2.35	1.96	Pull off
	198.22		32.59		1.65		Pull off
	205.09		33.72		1.87		Pull off
GFRP-22-5D	252.33	244.65	33.19	32.18	1.65	1.48	Pull off
	244.43		32.15		1.23		Pull off
	237.20		31.20		1.56		Pull off
GFRP-22-6D	271.03	267.61	32.34	31.25	0.86	1.02	Rebar fracture
	266.85		30.16		1.25		Rebar fracture
	264.95		31.26		0.95		Rebar fracture
GFRP-24-5D	292.06	284.97	32.28	31.50	1.66		Pull off
	285.01		31.50		1.27	1.59	Pull off
	277.86		30.71		1.83		Pull off
GFRP-24-6D	328.54	325.91	30.26	31.01	0.75	1.13	Rebar fracture
	322.10		31.77		1.12		Rebar fracture
	327.08		30.99		1.53		Rebar fracture

## Data Availability

The original contributions presented in the study are included in the article, further inquiries can be directed to the main authors.
